# Clinical Outcomes and Complications After INTACS Implantation in Keratoconus: A Systematic Review

**DOI:** 10.3390/jcm15114076

**Published:** 2026-05-25

**Authors:** Wiktoria Czuj-Porębska, Adam Wylęgała, Anna Martyka, Natalia Gospodarczyk, Jarosław Piłat, Edward Wylęgała, Bogumił Wowra

**Affiliations:** 1 Department of Ophthalmology, District Railway Hospital in Katowice, Medical University of Silesia, 40-760 Katowice, Poland; 2 Experimental Ophthalmology Unit, Department of Biophysics, Railway Hospital in Katowice, Medical University of Silesia, 40-760 Katowice, Poland; 3 Department of Ophthalmology, Faculty of Medical Sciences, School of Medicine in Zabrze, Medical University of Silesia, 40-760 Katowice, Poland

**Keywords:** corneal parameters, complications, intracorneal ring, INTACS, keratoconus, visual outcomes

## Abstract

**Background/Objectives**: Keratoconus is a progressive corneal ectatic disorder leading to irregular astigmatism and visual impairment. INTACS intracorneal ring segments are used to improve corneal shape and visual function; however, postoperative complications may occur. A comprehensive and evidence-based evaluation of visual outcomes and complications after INTACS implantation is therefore warranted. **Methods**: PubMed, Scopus, and Google Scholar were searched for English-language articles published between January 2015 and December 2025 using the terms INTACS, intracorneal ring segments, keratoconus, and complications, following a systematic literature search conducted in accordance with PRISMA 2020 guidelines. Only studies reporting INTACS-specific outcomes were included. Studies evaluating other intracorneal ring systems were excluded unless INTACS data could be separately extracted. **Results**: Seventeen studies comprising 544 eyes were included. INTACS implantation was associated with consistent improvements in visual acuity and corneal parameters. However, clinically relevant device-related complications, including segment migration, extrusion, and the need for secondary procedures such as repositioning or explantation, remain an important limitation. These findings indicate that although INTACS is an effective corneal regularization strategy, long-term safety depends on careful patient selection, precise surgical technique, and close postoperative surveillance. **Conclusions**: INTACS implantation is an effective option for visual rehabilitation in patients with keratoconus; however, its long-term safety is limited by the risk of device-related complications. Careful patient selection, appropriate surgical technique, and structured postoperative follow-up are essential to optimize outcomes and minimize adverse events.

## 1. Introduction

Keratoconus is a bilateral, asymmetric corneal ectatic disorder characterized by progressive stromal thinning and corneal protrusion, leading to irregular astigmatism and reduced visual acuity. The disease usually manifests in the second to third decade of life and progresses over time, significantly affecting visual function and quality of life [1]. Epidemiological studies report a global prevalence ranging from approximately 1:375 to 1:2000, with higher prevalence observed in Middle Eastern and Asian populations [2,3].

The etiology of keratoconus is multifactorial, involving genetic susceptibility, environmental and behavioral factors such as eye rubbing and atopy, and molecular mechanisms affecting the corneal extracellular matrix. Oxidative stress, keratocyte dysfunction, and altered collagen architecture contribute to progressive biomechanical weakening and corneal ectasia [4,5], limiting the effectiveness of conservative optical correction in moderate and advanced disease stages [1,6].

Management of keratoconus has evolved substantially, with treatment strategies individualized to disease severity and progression. While spectacles and contact lenses remain first-line options in early stages, progressive disease often requires interventional therapies, including corneal collagen cross-linking (CXL), intracorneal ring segment (ICRS) implantation, or keratoplasty in advanced cases [2,3,7].

ICRS, such as the INTACS system, are implanted within the mid-peripheral corneal stroma to induce central corneal flattening and reduce corneal irregularity through an arc-shortening effect [8,9]. Clinical studies demonstrate improvements in visual acuity, refractive error, and corneal topography following INTACS implantation [9,10]. However, long-term outcomes remain variable, with reports of refractive regression despite sustained improvements in corrected distance visual acuity [9].

Clinical studies have demonstrated overall benefits of ICRS implantation; however, substantial heterogeneity of reported outcomes, limited predictability, and the risk of postoperative complications remain [11]. Despite extensive literature describing visual and refractive outcomes after ICRS implantation, evidence specifically focused on INTACS-related complications, explantation, and long-term safety remains comparatively limited and heterogeneous. Therefore, a comprehensive evaluation of clinical outcomes and complications following INTACS implantation is warranted. This review aims to synthesize the current evidence regarding the efficacy and safety of INTACS implantation in keratoconus.

### 1.1. Keratoconus as a Clinical Problem

From a clinical perspective, keratoconus is associated with progressive visual deterioration caused by irregular astigmatism and corneal distortion. Patients frequently experience reduced visual acuity and difficulty achieving satisfactory spectacle correction, which may significantly impair visual function and quality of life [3,12]. The disease is typically bilateral but asymmetric, with variable severity between eyes [3,13]. In advanced stages, corneal scarring and acute corneal hydrops may occur, potentially leading to irreversible visual loss and the need for corneal transplantation [12,14].

A major clinical challenge is the diagnosis of early and subclinical disease, as structural and biomechanical alterations may precede overt clinical signs. Although advances in corneal imaging and biomechanical assessment have improved detection, variability in diagnostic criteria and disease staging remains a limitation in routine practice [15,16,17].

The chronic and unpredictable nature of keratoconus, combined with its long-term management requirements, underscores its relevance as an ongoing clinical problem and highlights the need for timely diagnosis, appropriate staging, and individualized therapeutic strategies [1,3,18].

### 1.2. Role of ICRS in the Treatment of Keratoconus

ICRS are small synthetic implants placed within the corneal stroma to modify corneal shape and refractive properties. Their implantation leads to flattening of the central cornea and improvement in corneal regularity, which may result in reduced irregular astigmatism and improved visual function. ICRS are therefore considered a surgical option in selected patients with keratoconus who continue to experience inadequate visual function or poor tolerance of contact lenses, but who do not yet require corneal transplantation. However, advances in scleral lens technologies, wider adoption of CXL, and the emergence of alternative approaches such as corneal allogenic intrastromal ring segments (CAIRS) may have contributed to a more limited and selective role of INTACS implantation in contemporary clinical practice. The concept of altering corneal refractive power by implanting a corneal ring was first introduced by Blevatskaya in 1966. Early designs consisted of a complete 360° intracorneal ring; however, this approach was associated with significant postoperative complications, particularly those related to wound healing at the incision site. As a result, full-ring designs were abandoned in favor of segmented implants, which demonstrated improved safety and clinical outcomes. The use of intracorneal ring segments specifically for the treatment of keratoconus was proposed by Joseph Colin in 2000 [19,20].

### 1.3. Types of ICRS

Several types of ICRS are currently available for the management of keratoconus, differing in design, optical zone diameter, arc length, and implantation technique [21,22,23]. The most commonly used synthetic polymethyl methacrylate (PMMA)-based systems include INTACS, KeraRing, Ferrara Ring, and MyoRing [21,22,23].

INTACS are implanted in the mid-peripheral cornea and are commonly used in mild to moderate keratoconus. KeraRing and Ferrara Ring systems are available in multiple arc lengths and thicknesses, allowing for more customized treatment of asymmetric cones and irregular astigmatism [24,25,26]. MyoRing represents a full 360° intracorneal ring design implanted into a stromal pocket [21,26].

More recently, biologic implants such as CAIRS have emerged as a potential alternative approach for selected patients with keratoconus [27].

### 1.4. INTACS—Mechanism of Action

The primary mechanism of action of INTACS is biomechanical rather than refractive. By adding volume to the peripheral cornea, INTACS induce a shortening of the arc length of the corneal lamellae, resulting in central corneal flattening and regularization of corneal curvature [28,29].

The procedure also redistributes biomechanical stress within the cornea and may improve corneal symmetry. INTACS implantation is tissue-sparing and reversible as the segments can be removed or exchanged if necessary. Femtosecond laser-assisted channel creation may improve surgical precision by allowing more accurate control of implantation depth and centration [28,30].

### 1.5. Clinical Application

INTACS do not halt the progression of keratoconus; therefore, they are frequently combined with CXL to achieve both biomechanical stabilization and optical rehabilitation. This combined approach has been shown to enhance long-term visual and topographic outcomes [20,28,31].

The primary clinical indication for INTACS implantation is mild to moderate keratoconus with documented visual deterioration and contact lens intolerance. Numerous studies have demonstrated that ICRS implantation leads to significant improvements in uncorrected and corrected distance visual acuity, reductions in spherical equivalent, and decreases in maximum keratometry values [28,29,32].

INTACS are also indicated in cases of iatrogenic corneal ectasia following refractive surgery. In these patients, ICRS can partially restore corneal shape, reduce irregular astigmatism, and improve functional vision. Clinical outcomes are generally comparable to those observed in keratoconus, provided that sufficient corneal thickness is present [20,29].

By regularizing the corneal surface, INTACS often improve contact lens tolerance, allowing patients to resume rigid gas-permeable or scleral lens wear. In some cases, the improvement in corneal symmetry enables satisfactory spectacle correction, delaying or avoiding the need for corneal transplantation [33].

## 2. Methods

### 2.1. Study Design and Reporting Standards

This systematic review was conducted in accordance with the PRISMA 2020 guidelines (PRISMA 2020 Checklist in the Supplementary Materials) Due to heterogeneity among the included studies, a quantitative synthesis (meta-analysis) was not performed, and results were synthesized narratively. The review was not registered in the PROSPERO database, and no predefined protocol was developed. The absence of prospective registration constitutes a methodological limitation and may increase the risk of selective reporting bias. To minimize this risk, predefined eligibility criteria were applied, and study selection, data extraction, and risk of bias assessment were performed independently by multiple reviewers, with discrepancies resolved through consensus.

### 2.2. Search Strategy

An electronic literature search was conducted using the PubMed, Scopus, and Google Scholar databases. The final literature search was conducted in January 2026 and included studies indexed up to 31 December 2025. The search was limited to English-language articles published between January 2015 and December 2025. The following search strategy was used with database-specific adaptations: (“intracorneal ring segments” OR INTACS OR “intrastromal corneal ring”) AND (complications OR “adverse effects”) AND keratoconus. In Google Scholar, publication years were limited to 2015–2025, and English-language studies were selected manually during screening because language filters were not available. Reference lists of relevant studies were additionally screened to identify potentially eligible articles. Complete database-specific search strategies are provided in Supplementary Material—Search String.

### 2.3. Eligibility Criteria

Studies were eligible if they reported clinical outcomes or complications following INTACS implantation in keratoconus. Review articles, editorials, conference abstracts, and non-English publications were excluded. Studies evaluating other types of intracorneal ring segments (for example, Keraring, Ferrara, and MyoRing) were excluded unless INTACS-specific data could be separately extracted. Although broader terms such as “intracorneal ring segments” and “intrastromal corneal ring” were included in the search strategy to ensure sensitivity, only studies reporting outcomes specifically for INTACS devices were included in the final analysis.

### 2.4. Study Selection

**Figure 1:**
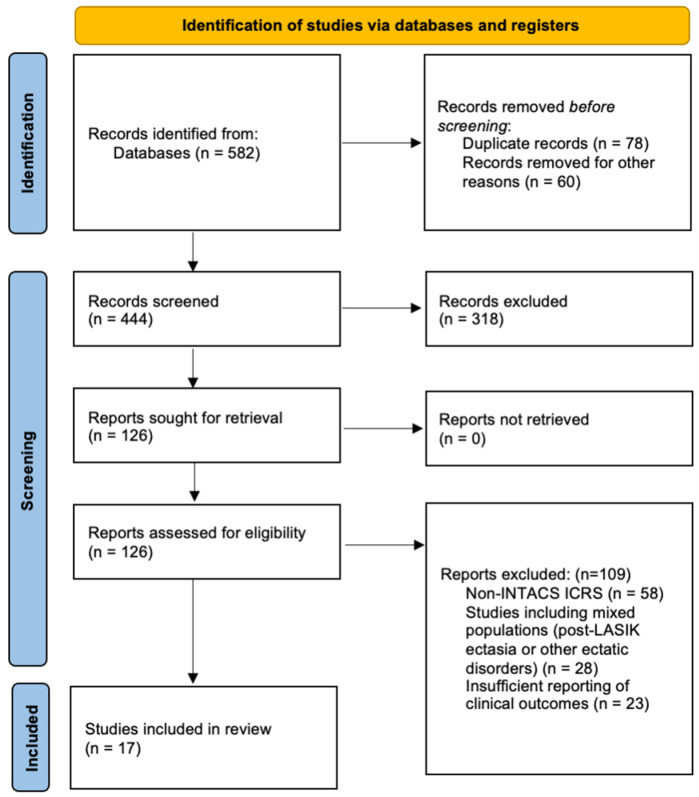
PRISMA flow diagram of the study selection process.

Title and abstract screening, as well as full-text assessment for eligibility, were performed independently by two reviewers (W.C.-P. and A.M.). Any discrepancies were resolved through discussion; if agreement could not be reached, a third reviewer (N.G.) was consulted. The study selection process is illustrated in Figure 1 (PRISMA 2020 flow diagram).

### 2.5. Data Extraction

Data extraction was performed independently by two reviewers (W.C.-P. and A.M.) using a standardized approach. Any discrepancies were resolved through discussion and consensus. Extracted information included study characteristics (author, year of publication, and study design), patient-related data (number of eyes), details of the intervention, follow-up duration, visual and refractive outcomes, and reported postoperative complications.

### 2.6. Outcomes of Interest

The primary outcomes of interest were postoperative visual and refractive outcomes following INTACS implantation, including changes in uncorrected and corrected distance visual acuity. Secondary outcomes included the occurrence of early and late postoperative complications and safety-related events.

### 2.7. Risk of Bias Assessment

The methodological quality of the included studies was assessed independently by two reviewers (W.C.-P. and N.G.) using the Joanna Briggs Institute (JBI) Critical Appraisal Checklists appropriate for each study design (case series, cohort studies, quasi-experimental studies, and case reports). Discrepancies were resolved through discussion and consensus. Each study was evaluated using predefined checklist items, including participant selection, measurement of outcomes, completeness of follow-up, and appropriateness of statistical analysis. Based on the number of criteria met, an overall methodological quality rating was assigned to each study. Most studies were assessed as having moderate methodological quality, with common limitations including incomplete follow-up and lack of control groups. Detailed results of the critical appraisal are presented in Supplementary Tables S1–S4.

## 3. Results

Studies evaluating INTACS implantation as a standalone procedure reported moderate but clinically meaningful improvements in visual acuity and corneal keratometric parameters. Across the included studies, uncorrected distance visual acuity (UDVA) improved by approximately 2–4 Snellen lines, while corrected distance visual acuity (CDVA) showed smaller but significant gains. Reductions in Kmax and Kmean values were observed in most studies, indicating central corneal flattening and improved corneal regularity. However, long-term follow-up data suggest that visual benefits may diminish over time in selected patients, particularly in eyes with more advanced keratoconus. Despite initial improvement, some studies reported partial regression of UDVA several years after implantation.

Several studies evaluated the combination of INTACS implantation with CXL to achieve both optical improvement and biomechanical stabilization. Both simultaneous and sequential treatment protocols were reported. Across studies, combined treatment was associated with sustained improvements in visual acuity and keratometric parameters during follow-up, with greater refractive stability compared with INTACS implantation alone. However, combined procedures may be associated with a higher incidence of complications due to increased surgical complexity. Direct comparisons between simultaneous and sequential protocols were limited by heterogeneity in study design.

Studies comparing femtosecond laser-assisted and manual intrastromal channel creation techniques reported comparable visual and keratometric outcomes following INTACS implantation. However, femtosecond laser-assisted techniques were consistently associated with a lower incidence of mechanical complications, including segment migration and extrusion, reflecting greater surgical precision. Overall, differences between techniques were more pronounced in safety profiles than in refractive efficacy. Several studies restricted INTACS implantation to eyes with a minimum corneal thickness of approximately 400 µm and without central corneal scarring, reflecting commonly applied eligibility criteria.

### 3.1. Study Characteristics

The characteristics of the included studies are summarized in Table 1. Changes in visual acuity and keratometric parameters reported across the included studies are summarized in Table 2.

### 3.2. Visual and Refractive Outcomes

Visual and refractive outcomes following INTACS implantation are summarized in the studies included in this review.

### 3.3. Clinical Magnitude of Effect

INTACS and INTACS SK implantation resulted in a moderate but clinically meaningful improvement in visual acuity. UDVA improved by approximately 0.28–0.43 logMAR (about 2–4 Snellen lines), while CDVA improved by 0.04–0.27 logMAR (about 0.5–2 Snellen lines). A clinically significant gain (≥1 Snellen line) was observed in the majority of treated eyes. However, long-term follow-up data reported by Kang et al. [9] demonstrated that UDVA improvement may diminish over time, with visual acuity worsening at 5 years compared to earlier postoperative visits, approaching preoperative values.

Several studies indicate that the magnitude of visual improvement after INTACS implantation is reduced in more advanced forms of keratoconus. Better outcomes are consistently reported in eyes with mild to moderate disease, whereas eyes with advanced keratoconus demonstrate smaller and more variable gains in visual acuity and refractive parameters.

Very steep preoperative keratometry values (high Kmax) have been identified as an important limiting factor. Some studies excluded eyes with high Kmax values, which suggests that excessive corneal steepness is associated with reduced predictability and smaller visual benefit after INTACS implantation.

The presence of corneal scarring is another factor associated with poor visual outcomes. Several studies excluded eyes with corneal scars, underscoring that stromal opacity and disrupted collagen architecture limit the functional improvement.

**Figure 2:**
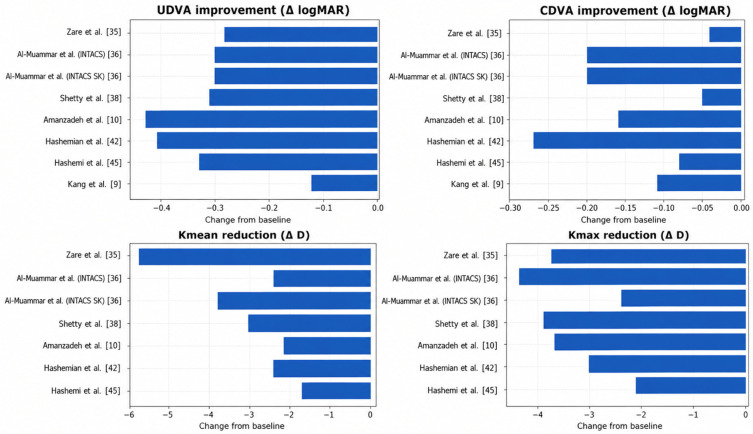
Summary of reported visual and topographic outcomes following INTACS implantation in keratoconus. Horizontal bar charts present the magnitude of change from baseline reported across included studies for UDVA, CDVA, Kmax, and Kmean. Negative values indicate improvement in visual acuity parameters and reduction in keratometric measurements. Only studies with extractable quantitative data were included. Kang et al. [9], Amanzadeh et al. [10], Zare et al. [35], Al-Muammar et al. [36], Shetty et al. [38], Hashemian et al. [42], Hashemi et al. [45].

To improve the readability and quantitative synthesis of the reported outcomes, a graphical summary of changes in visual acuity and keratometric parameters across studies was added (Figure 2). The reported improvements in UDVA and CDVA, together with reductions in Kmax and Kmean, are summarized graphically to facilitate comparison between studies despite methodological heterogeneity.

### 3.4. Complications After INTACS Implantation

Reported complications after INTACS implantation were heterogeneous across studies and ranged from mild visual symptoms to severe mechanical and infectious events requiring explantation. Segment extrusion, migration, infectious keratitis, and visual deterioration represented the most frequently reported causes of secondary intervention or device removal. Reported explantation rates varied substantially between studies because of differences in follow-up duration, patient selection, and indication for device removal.

Complications following INTACS and INTACS SK implantation are relatively uncommon and can be classified according to timing (intraoperative, early, late complications), underlying mechanism (mechanical, infectious, inflammatory), and the need for reintervention. For clinical purposes, intraoperative and early postoperative complications are defined as events occurring during surgery or within the first three months after implantation. Late complications develop beyond this period, sometimes several years later.

Intraoperative complications are rare and predominantly mechanical. In a prospective series by Zare et al. [35], aqueous leakage during tunnel opening occurred in 2 of 32 eyes. Both cases were managed by immediate wound closure with sutures. One eye was scheduled for rechanneling, and the other eye for lamellar keratoplasty due to severe corneal thinning.

Early postoperative complications are infrequent and include mechanical, inflammatory and infectious events. Moshirfar et al. [39] described a delayed perforation occurring one week after surgery, attributed to excessive implantation depth, which was successfully managed by explantation.

Early postoperative infectious keratitis has been reported as a rare complication after ring implantation. Tabatabaei et al. [40] described two early cases of Gram-positive cocci keratitis associated with INTACS; however, device-specific incidence rates could not be calculated due to the inclusion of multiple ICRS types. Additional early infectious events were reported by Hersh et al. [49] in the setting of combined procedures, including keratitis occurring three days after simultaneous INTACS implantation and CXL, as well as one case developing one week after CXL performed following prior INTACS implantation. In the first case, infectious keratitis was diagnosed as Staphylococcus aureus and was successfully treated with antibacterial therapy, leaving residual corneal haze. In the second case, fungal keratitis caused by Penicillium species required antifungal treatment and resulted in residual corneal scarring. Hersh et al. [49] also described early postoperative inflammation around the INTACS in three eyes (two occurring after sequential CXL following prior ICRS implantation and one after ICRS implantation alone) with INTACS explantation required in two of these cases. Additionally, glare symptoms were reported in one eye after concurrent ICRS implantation and CXL, and the segments were removed.

In a large retrospective series of 572 eyes, Nguyen et al. [50] reported several postoperative adverse events following INTACS implantation. Microbial keratitis occurred in 1/572 eyes, presenting 6 weeks after surgery and complicated by segment extrusion and corneal perforation, which required urgent ICRS explantation with intensive topical antibiotic therapy; despite resolution, a residual corneal scar remained. Sterile inflammatory infiltrates around the segment were observed in 11/572 eyes, including three cases with stromal melting and thinning. Persistent symptoms also led to device removal in selected cases, including foreign-body sensation (2/572) and persistent photophobia (1/572). In addition, 20 eyes underwent INTACS explantation due to refractive or topographic considerations. Apart from microbial keratitis presenting 6 weeks after implantation, the time of onset for the remaining complications was not specified.

Segment extrusion is a recognized late complication after INTACS implantation. Abreu et al. [34] reported segment extrusion in 1/14 eyes, occurring 7 months postoperatively; no surgical intervention was required, and the patient maintained satisfactory visual acuity.

Al-Habboubi et al. [41] analyzed explanted intracorneal ring segments (ICRS) in a cohort in which INTACS SK and INTACS constituted the vast majority of removed devices (>90%), making the findings highly relevant to INTACS-based procedures. Two groups were compared based on the location of the primary surgery: group one included eyes in which implantation and explantation were performed at the same institution (n = 41) whereas group two comprised eyes implanted elsewhere and referred for explantation (n = 29). In group one, the leading indications for segment removal were visual disturbances (45.2%), followed by segment extrusion (38.1%)**,** corneal neovascularization (9.1%), and infectious keratitis (7.1%). In the referral cohort, extrusion was the most common reason for explantation (41.4%), followed by visual disturbances (27.5%), infection (17.2%), and neovascularization (13.7%). The mean time from implantation to explantation was 29.6 months in group one and 37.3 months in group two; 76% of explantations occurred within the first four years and 60% of extrusion cases occurred during the first year.

Zare et al. [35] reported late segment migration with corneal melting and extrusion in 2/32 eyes, which was managed by explantation. Corneal deposits adjacent to the ring channel occurred in 5/32 eyes at 6 months and did not require treatment. Hashemian et al. [42] also mentioned minor peri-segment deposits in two eyes, which did not require treatment.

In a retrospective explantation series by Chhadva et al. [44], INTACS removal was performed in all eyes due to functional visual deterioration, including worsening visual acuity alone (8/10 eyes) or worsening visual acuity associated with segment overlap (2/10 eyes). In the overlap subgroup, explantation was performed at approximately 54 weeks after implantation. Despite explantation, four patients subsequently required penetrating keratoplasty due to progressive visual deterioration.

In long-term outcome studies, reported complication rates may be influenced by case selection. In the five-year series by Kang et al. [9], eyes with significant complications were excluded from the final analysis, and no additional complications were observed in the remaining cohort during follow-up. Of the 87 eyes initially treated with INTACS, four were excluded due to complications, including one ring extrusion, two inflammatory infiltrations around the segments, and one case of corneal opacity; the timing of these events was not specified.

Abad et al. [37] reported anterior stromal necrosis (ASN) as a very late complication, observed in 9/98 eyes. The mean time between implantation and ASN diagnosis was 10.5 ± 1.3 years. The condition occurred predominantly in case of inferotemporally placed segments (8/9 eyes), with one case involving a temporal segment. Segment explantation was performed in 7/9 eyes, in the remaining two eyes, explantation was not carried out because one patient declined surgery and another could not proceed due to insurance-related issues.

It should be noted that very late and severe complications have also been described in eyes treated with INTACS for post-LASIK ectasia. Moshirfar et al. [51] reported acute corneal hydrops caused by posterior migration of an INTACS segment into the anterior chamber 7 years after implantation, requiring explantation.

Although outside the primary 2015–2025 time frame, earlier publications have described rare but severe complications following INTACS implantation that remain clinically relevant. Mitchell et al. [52] reported a case of fungal keratitis caused by Candida parapsilosis, which required prolonged antifungal therapy and ultimately therapeutic keratoplasty due to progressive corneal involvement. Similarly, Barbara et al. [53] described progressive corneal neovascularization and lipid keratopathy developing after INTACS SK implantation, complicated by stromal melting and corneal perforation, necessitating segment explantation and subsequent penetrating keratoplasty.

**Figure 3:**
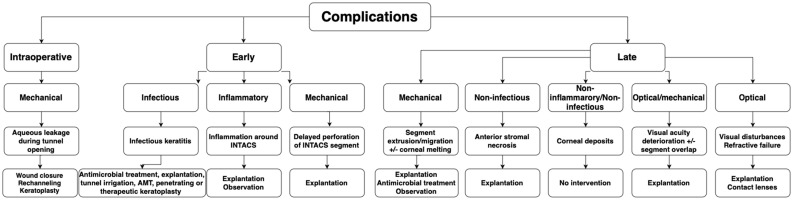
Complications management algorithm after INTACS/INTACS SK implantation. Complications are categorized according to the time of onset and underlying mechanism. The algorithm is based on the findings of the present review.

Based on the reported complications and management strategies, a conceptual management algorithm was developed to summarize the findings (Figure 3).

#### Corneal Tissue Response After INTACS Implantation

INTACS implantation can induce stromal remodeling even without clinically significant complications. Hamon et al. described two main tissue patterns: peri-segmental fibrosis and lamellar channel deposits, documented with slit-lamp examination and in vivo confocal microscopy. These structural changes were not associated with clinically relevant refractive and topographic consequences [54].

Al-Amry et al. reported a histopathological case with intrastromal deposits along the INTACS channels containing CD68-positive foamy histiocytes, which were not clinically visually significant [55]. Kapelushnik et al. [56] confirmed ex vivo that peri-channel whitish deposits after INTACS are accompanied by minimal keratocyte fibroblastic change without inflammation, supporting the concept of a tissue response to the implant rather than the surgical technique.

Long-term ultrastructural changes have also been demonstrated in explanted post-INTACS keratoconus tissue, including lamellar disorganization, reduced collagen fibril diameter, and altered proteoglycan distribution, suggesting chronic extracellular matrix remodeling in selected eyes [57].

## 4. Discussion

This review evaluated visual outcomes, keratometric changes, and postoperative complications following INTACS implantation in patients with keratoconus. Overall, the analyzed studies demonstrated that INTACS implantation is associated with an improvement in both uncorrected and corrected distance visual acuity, accompanied by a reduction in corneal keratometric parameters, particularly maximum keratometry. These findings support the role of ICRS as an effective option for visual rehabilitation in selected patients with keratoconus. Nevertheless, substantial heterogeneity in reported outcomes was observed across studies, reflecting differences in patient characteristics, disease severity, surgical technique, and adjunctive treatments. Visual improvement after INTACS implantation appears to be primarily related to corneal shape regularization rather than full refractive correction [58]. Even moderate flattening of the cornea may significantly reduce irregular astigmatism and higher-order aberrations, resulting in meaningful functional visual gains despite residual refractive error. This observation is consistent across multiple observational series included in this review. Despite the improvement in corneal regularity following intracorneal ring segment implantation, optimal visual acuity is not always achieved with the procedure alone. Several studies have demonstrated that contact lenses may still be required for visual rehabilitation in selected patients after ICRS implantation. Fernández-Velázquez and Fernández-Fidalgo reported that custom-made silicone hydrogel contact lenses could be successfully fitted in keratoconus patients previously treated with ICRS, leading to further improvement in visual outcomes [59]. Similarly, scleral contact lenses represent an effective adjunctive option for visual correction and improved comfort in the presence of residual irregular astigmatism after ICRS implantation [60,61]. Postoperative complications, although relatively infrequent, remain a clinically relevant concern. Reported adverse events ranged from mild symptoms such as glare or foreign body sensation to more serious complications including infectious keratitis, segment migration or extrusion, corneal melting, and the need for secondary surgical intervention. A key factor influencing complication rates is the method of intrastromal channel creation. Evidence indicates that femtosecond laser-assisted channel creation is associated with a significantly lower complication rate compared with manual mechanical dissection. In the comparative study by Monteiro et al., complications occurred in 18.1% of eyes in the manual group versus 3.6% in the femtosecond laser group [62]. This difference is largely attributed to the greater precision and predictability of femtosecond laser-assisted tunnel creation, which allows for more accurate control of implantation depth and centration, thereby reducing the risk of superficial or asymmetric segment placement. Importantly, visual and refractive outcomes appear broadly comparable between techniques in uncomplicated cases, suggesting that the primary advantage of femtosecond laser use lies in improved safety rather than superior efficacy. Long-term corneal response to INTACS implantation must also be considered. Ultrastructural studies have demonstrated stromal alterations years after implantation, including disruption of lamellar organization and changes in collagen fibril architecture, which may contribute to late visual deterioration in selected patients [57]. These findings support the concept that INTACS do not halt the ectatic process and emphasize the potential benefit of combining INTACS implantation with CXL to achieve both optical rehabilitation and biochemical stabilization.

### 4.1. Clinical Implications

From a practical clinical perspective, INTACS represents a useful but limited option in the management of keratoconus. Based on the available evidence, ICRS implantation should be regarded primarily as a visual rehabilitation procedure rather than a treatment that modifies the natural course of the disease. Although improvements in corneal shape and visual performance are frequently observed, progression of ectasia may still occur, particularly in younger patients. For this reason, the role of INTACS in real-world clinical practice is best confined to carefully selected patients with mild to moderate keratoconus who are unable to achieve satisfactory vision with spectacles or contact lenses and who are not yet candidates for corneal transplantation.

In appropriately selected cases, INTACS implantation may provide clinically meaningful visual improvement and delay the need for more invasive surgical procedures. However, these potential benefits must be balanced against the risks associated with implantation and the realistic expectations of both patients and clinicians. Long-term data indicate that a substantial proportion of patients continue to require contact lenses after surgery, emphasizing that ICRS implantation does not replace optical correction but may improve corneal regularity and lens tolerance. From a practical and economic perspective, this limits the procedure to situations in which the anticipated functional gain justifies the surgical risk, cost, and need for ongoing follow-up.

Several factors continue to limit wider adoption of INTACS implantation. Access to femtosecond laser technology, which is associated with improved surgical precision and lower complication rates compared with manual techniques, remains uneven across healthcare systems. In addition, surgical experience plays a critical role, with higher complication rates reported during the learning curve. These factors limit the standardization of outcomes and confine the safe use of INTACS implantation primarily to centers with sufficient experience and technical resources.

Another important limitation is the lack of widely accepted nomograms and clear patient selection criteria. In everyday practice, treatment decisions often depend on individual surgeon experience, which inevitably leads to variability in outcomes and may increase the likelihood of late complications or the need for explantation. In addition, published studies differ considerably in how outcomes are reported. While short-term improvements in visual acuity or keratometric parameters are commonly described, long-term safety, functional vision, explantation rates, and patient-reported outcomes are reported less consistently. This variability makes meaningful comparison between studies difficult and limits the applicability of current evidence to clinical guideline development.

### 4.2. Future Perspectives

Future improvements in this field should focus on improved patient stratification and more complete assessment of outcomes. Including factors such as corneal biomechanics, cone location, disease activity, and individual visual needs in preoperative planning may help better identify patients who are most likely to benefit from ICRS implantation. In addition, more standardized outcome reporting, with greater emphasis on long-term complications, quality of life, and ongoing need for contact lenses, would allow for a more realistic evaluation of the clinical value of this procedure. Further refinement of planning methods may contribute to safer and more consistent results, although stronger long-term data are still needed.

Further research is still needed as studies limited to short-term observations only provide some insight and should be complemented by analyses focused on clinically meaningful outcomes. In particular, studies assessing long-term visual function, stability of visual improvement, and comparisons between different treatment strategies, including combined treatment with CXL, are important. Given the chronic and variable course of keratoconus, it is unlikely that a single, definitive study endpoint can be defined.

In the coming years, the role of INTACS is expected to become increasingly restricted and directed toward a clearly defined subset of patients in whom meaningful functional benefits cannot be achieved using less invasive or more predictable treatment modalities, including increasingly customized and patient-specific contact lens designs. The increasing availability of alternative treatment strategies, including CAIRS, advanced scleral lens technologies, and combined CXL-based approaches, may reduce the current indications for INTACS implantation and restrict its use to carefully selected patients with keratoconus.

### 4.3. Limitations

This review is limited by heterogeneity among the included studies in terms of study design, patient selection, surgical technique, and follow-up duration. Most included studies were observational case series, and most studies were of moderate methodological quality, reflecting the limited availability of randomized controlled trials in this field. Variations in the reporting of visual and keratometric outcomes, along with differences in the application and timing of combined procedures such as CXL, limited direct comparison between studies. In addition, long-term complication rates may be underestimated because of incomplete follow-up in several reports. In addition, the predominance of non-randomized studies limits the overall strength of the evidence.

## 5. Conclusions

INTACS implantation provides effective visual and topographic improvement in appropriately selected patients with keratoconus. Available evidence suggests that femtosecond laser-assisted channel creation may be associated with lower rates of mechanical and late postoperative complications compared with manual techniques, although most included studies were observational and heterogeneous. Careful patient selection, precise surgical technique, and long-term follow-up remain important in optimizing outcomes and minimizing adverse events. Further studies with standardized outcome reporting and longer follow-ups are needed to better define the long-term efficacy and safety of INTACS implantation.

## Figures and Tables

**Table 1:** Characteristics of Included Studies.

Author	Study	Comparison Group	Intervention	Eyes (n)	Mean Age (Years)	Male (%)	ICRS Type	Channel Creation	Follow-Up
Abreu et al. (2018) [34]	Retrospective case series	Pre-INTACS baseline	INTACS implantation	14	15.36 ± 2.84	64.3	INTACS INTACS SK	Mechanical dissection	6.36 ± 0.97 y
Zare et al. (2016) [35]	Prospective interventional case series	Pre-INTACS baseline	INTACS implantation	32	23.8 ± 5.4	56	INTACS SK	Femtosecond laser-assisted	6 m
Al Muammar et al. (2015) [36]	Retrospective case series	INTACS vs. INTACS SK	INTACS implantation	INTACS: 16 INTACS SK: 18	INTACS: 29.6 ± 5.4 INTACS SK: 28.1 ± 4.9	INTACS: 76.9 INTACS SK: 30.3	INTACS INTACS SK	Mechanical dissection	12 m
Abad et al. (2020) [37]	Consecutive interventional case series	None	Management of long-term INTACS complications	9	NR (ASN cases)	NR (ASN cases)	INTACS	Mechanical dissection	10.5 ± 1.3 y
Shetty et al. (2021) [38] (INTACS-only data extracted)	Prospective interventional case series	Pre-INTACS baseline	INTACS followed by TRESK + accelerated CXL	48	23.39 ± 0.62	64.4	INTACS	Femtosecond laser-assisted	1 m post-INTACS, pre-TRESK/CXL
Amanzadeh et al. (2017) [10]	Prospective interventional case series	Pre-INTACS baseline	INTACS implantation	42	30.5 ± 8.11	76.2	Single segment INTACS	Femtosecond laser-assisted	4 m
Moshirfar et al. (2021) [39]	Case report	None	INTACS explantation	1	35	0	INTACS	NR	3 weeks after explantation
Tabatabaei et al. (2019) [40] (INTACS-only data extracted)	Retrospective case series	None	Management of microbial keratitis after ICRS implantation	2	24	100	INTACS	Femtosecond laser-assisted, Mechanical dissection	6 m after keratitis treatment
Al-Habboubi et al. (2022) ^1^ [41] (INTACS-only data extracted)	Retrospective cohort	None	INTACS explantation	64	NR	NR	INTACS INTACS SK	Femtosecond laser-assisted, Mechanical dissection	29.6–37.3 m (time to explantation)
Hashemian et al. (2018) [42]	Prospective interventional case series	Pre-INTACS baseline	INTACS implantation	71	27.5 ± 7.1	63.5	INTACS SK	Femtosecond laser-assisted	6 m
Bteich et al. (2025) [43]	Comparative case series	INTACS-SK (6 mm vs. 7 mm)	INTACS implantation	12 Group 1 = 6 Group 2 = 6	NR	NR	INTACS SK (6.0 and 7.0 mm)	Femtosecond laser-assisted	6 m
Chhadva et al. (2015) [44]	Retrospective case series	None	INTACS explantation	10	38 ± 11	50	INTACS	NR	1 m after explantation
Hashemi et al. (2015) [45]	Prospective interventional case series	None	INTACS implantation	62	29.48 ± 7.21	61.3	Single segment INTACS	Femtosecond laser-assisted	12 m
Kang et al. (2019) [9]	Retrospective cohort	None	INTACS implantation	30	27.69 ± 6.68	61	INTACS	Femtosecond laser-assisted	5 y
Koh et al. (2019) [46]	Retrospective interventional case series	Pre-INTACS baseline	INTACS implantation (first stage of two-stage protocol)	30	27 ± 6	68	INTACS SK	Femtosecond laser-assisted	3 m after INTACS-SK implantation (before PRK-CXL)
Flockerzi et al. (2024) [47]	Retrospective longitudinal case series	Pre-INTACS baseline	INTACS implantation	49	31 ± 10	78	INTACS SK	Femtosecond laser-assisted	10.6 ± 2.3 m
Rho et al. (2016) [48]	Retrospective case series	None	INTACS implantation	34	27.0 ± 6.7	64.7	INTACS SK	Femtosecond laser-assisted	3 m

NR—not reported; ASN—anterior stromal necrosis; TRESK—topography-guided removal of epithelium and stroma with keratectomy; m—month; y—year. For studies including multiple types of intracorneal ring segments, only eyes treated with INTACS were counted and reported in this table. The follow-up time reported corresponds to the time point used for outcome extraction in Table 2. ^1^ For Al-Habboubi et al., the number of INTACS-treated eyes was estimated from percentages reported by the authors. The included studies were predominantly observational, consisting mainly of retrospective and prospective case series, with only a limited number of comparative studies and isolated case reports. The number of analyzed eyes varied widely, and study populations were generally composed of young to middle-aged adults with a consistent male predominance. Channel creation was most frequently performed using femtosecond laser assistance, while mechanical dissection was reported less often. Follow-up duration ranged from short term to long term, reflecting substantial heterogeneity in study design and outcome assessment.

**Table 2:** Changes in visual acuity and corneal keratometry before and after INTACS implantation.

Author	Follow-Up	UDVA (logMAR)			CDVA (logMAR)			Kmax (D)			Kmean (D)		
		Baseline	Follow-Up	Δ	Baseline	Follow-Up	Δ	Baseline	Follow-Up	Δ	Baseline	Follow-Up	Δ
Abreu et al. (2018) ^1^ [34]	6.36 ± 0.97 y	NR	NR	NR	NR	NR	NR	55.92 ± 4.56	52.19 ± 3.6	−3.73	NR	NR	NR
Zare et al. (2016) [35]	6 m	0.81 ± 0.3	0.53 ± 0.2	−0.28 ± 0.07	0.37 ± 0.2	0.32 ± 0.2	−0.04 ± 0.06	54.57 ± 3.9	49.73 ± 4.4	−4.35 ± 2.07	50.50 ± 2.9	44.62 ± 3.0	−5.73 ± 1.64
Al-Muammar et al. (2015) -INTACS [36]	6 m	0.8 ± 0.3	0.5 ± 0.3	−0.3 ± 0.2	0.4 ± 0.2	0.3 ± 0.2	−0.2 ± 0.2	47.7 ± 2.2	45.3 ± 2.8	−2.4 ± 2.4	45.7 ± 1.7	43.3 ± 2.4	−2.4 ± 2.0
Al-Muammar et al. (2015) -INTACS SK [36]	6 m	0.7 ± 0.4	0.4 ± 0.4	−0.3 ± 0.3	0.3 ± 0.2	0.1 ± 0.1	−0.2 ± 0.2	49.4 ± 2.9	45.5 ± 3.2	−3.9 ± 2.0	47.7 ± 2.8	43.9 ± 2.8	−3.8 ± 2.0
Shetty et al. (2021) [38]	1 m post-INTACS	1.05 ± 0.05	0.74 ± 0.07	−0.31	0.31 ± 0.03	0.26 ± 0.03	−0.05	63.49 ± 1.45	59.81 ± 1.22	−3.68	52.73 ± 0.97	49.68 ± 0.89	−3.05
Amanzadeh et al. (2017) [10]	4 m	0.92 ± 0.35	0.49 ± 0.31	−0.43 ± 0.28	0.39 ± 0.15	0.23 ± 0.11	−0.16 ± 0.14	55.95 ± 5.23	52.93 ± 4.16	−3.02 ± 2.88	48.03 ± 2.80	45.87 ± 2.61	−2.16 ± 1.09
Hashemian et al. (2018) [42]	6 m	0.87 ± 0.26	0.46 ± 0.19	−0.41	0.55 ± 0.21	0.28 ± 0.17	−0.27	NR	NR	NR	49.67 ± 3.98	47.22 ± 3.91	−2.45
Hashemi et al. (2015) [45]	12 m	0.84 ± 0.48	0.51 ± 0.48	−0.33	0.29 ± 0.17	0.21 ± 0.20	−0.08	49.12 ± 3.45	47.02 ± 3.87	−2.10	47.22 ± 3.46	45.30 ± 3.56	−1.92
Kang et al. (2019) [9]	5 y	0.857 ± 0.456	0.741 ± 0.372	−0.12	0.516 ± 0.298	0.404 ± 0.299	−0.11	NR	NR	NR	NR	NR	NR

NR—not reported; m—month; y—year; UDVA—uncorrected distance visual acuity; CDVA—corrected distance visual acuity; Kmax—maximum keratometry; Kmean—mean keratometry; logMAR—logarithm of the minimum angle of resolution. In one study, visual acuity improvement was reported qualitatively as a gain of 1–2 Snellen lines, without providing quantitative logMAR values; therefore, these data could not be included in the numerical comparison. When Δ (change from baseline) values were not explicitly reported, Δ values were calculated as the difference between postoperative and preoperative mean values. Negative Δ values indicate improvement. ^1^ Preoperative and postoperative UCVA and BCVA are in decimal scale.

## Data Availability

Data sharing is not applicable to this article as no new data were created or analyzed.

## Supplementary Materials

The following supporting information can be downloaded at: https://www.mdpi.com/article/10.3390/jcm15114076/s1. Figures S1–S4: Clinical documentation of INTACS-related complications. Tables S1–S4: JBI critical appraisal assessment for included studies. PRISMA 2020 Checklist. The PRISMA 2020 statement is cited in the reference list [63].

## Abbreviations

The following abbreviations are used in this manuscript:

ASN	anterior stromal necrosis
CAIRS	corneal allogenic intrastromal ring segments
CDVA	corrected distance visual acuity
CXL	corneal cross-linking
ICRS	intracorneal ring segments
JBI	Joanna Briggs Institute
Kmax	maximum keratometry
Kmean	mean keratometry
logMAR	logarithm of the minimum angle of resolution
NR	not reported
PMMA	polymethyl methacrylate
PRK	photorefractive keratectomy
TRESK	topography-guided removal of epithelium and stroma with keratectomy
UDVA	uncorrected distance visual acuity
