# Supplementary material for: Clinical Outcomes and Complications After INTACS Implantation in Keratoconus: A Systematic Review
Source: J Clin Med. 2026 May 25;15(11):4076. doi: 10.3390/jcm15114076 (PMC13258055; doi:10.3390/jcm15114076)
Supplement: Supplementary file 1 [file jcm-15-04076-s001.zip › SUPPLEMENTARY MATERIAL TABLES S1-S4.pdf]

Joanna Briggs Institute (JBI) Critical Appraisal Checklist

Table S1 - Case Series

Study	Q1	Q2	Q3	Q4	Q5	Q6	Q7	Q8	Q9	Methodological Quality
Abreu et al. (2018)	Yes	Yes	Yes	No	No	Yes	Yes	Yes	Yes	Moderate
Zare et al. (2016)	Yes	Yes	Yes	Yes	Yes	Yes	Yes	Yes	Yes	High
Abad et al. (2020)	Yes	Yes	Yes	Yes	Yes	Yes	Yes	No	Yes	Moderate
Amanzadeh et al. (2017)	Yes	Yes	Yes	No	No	Yes	Yes	Yes	Yes	Moderate
Chhadva et al. (2015)	Yes	Yes	Yes	No	No	Yes	Yes	Yes	Yes	Moderate

Questions

Q1. Were there clear criteria for inclusion in the case series?
Q2. Was the condition measured in a standard, reliable way for all participants?
Q3. Were valid methods used for identification of the condition for all participants?
Q4. Did the case series have consecutive inclusion of participants?
Q5. Did the case series have complete inclusion of participants?
Q6. Was there clear reporting of the demographics of the participants in the study?
Q7. Was there clear reporting of clinical information of the participants?
Q8. Were the outcomes or follow-up results of cases clearly reported?
Q9. Was there clear reporting of the presenting site(s)/clinic(s) demographic information?

Table S2 - Cohort

Study	Q1	Q2	Q3	Q4	Q5	Q6	Q7	Q8	Q9	Q10	Q11	Methodological Quality
Tabatabaei et al. (2018)	Yes	Yes	Yes	Yes	Yes	Yes	Yes	Yes	Yes	Yes	Yes	High
Al-Habboubi et al. (2022)	Yes	Yes	Yes	Yes	No	Yes	Yes	Yes	No	No	Yes	Moderate
Al-Muammar et al. (2015)	Yes	Yes	Yes	Yes	Yes	Yes	Yes	Yes	No	No	Yes	Moderate
Bteich et al. (2025)	Yes	Yes	Yes	Yes	Yes	Yes	Yes	Yes	Yes	No	Yes	Moderate

Questions

Q1. Were the two groups similar and recruited from the same population?
Q2. Were the exposures measured similarly to assign people to both exposed and unexposed groups?
Q3. Was the exposure measured in a valid and reliable way?
Q4. Were confounding factors identified?
Q5. Were strategies to deal with confounding factors stated?
Q6. Were the groups/participants free of the outcome at the start of the study (or at the moment of exposure)?
Q7. Were the outcomes measured in a valid and reliable way?
Q8. Was the follow-up time reported and sufficient to be long enough for outcomes to occur?
Q9. Was follow-up complete, and if not, were the reasons to loss to follow-up described and explored?
Q10. Were strategies to address incomplete follow-up utilized?
Q11. Was appropriate statistical analysis used?

Table S3 - Quasi-experimental

Study	Q1	Q2	Q3	Q4	Q5	Q6	Q7	Q8	Q9	Methodological Quality
Shetty et al. (2021)	Yes	Yes	Yes	No	No	Yes	Yes	Yes	Yes	Moderate
Hashemian et al. (2018)	Yes	Yes	Yes	No	No	Yes	Yes	Yes	Yes	Moderate
Hashemi et al. (2015)	Yes	Yes	Yes	No	No	Yes	Yes	Yes	Yes	Moderate
Kang et al. (2019)	Yes	Yes	Yes	No	No	No	Yes	Yes	Yes	Moderate
Koh et al. (2019)	Yes	Yes	Yes	No	Yes	Yes	Yes	Yes	Yes	Moderate
Flockerzi et al. (2024)	Yes	Yes	Yes	No	No	No	Yes	Yes	Yes	Moderate
Rho et al. (2016)	Yes	Yes	Yes	No	No	Yes	Yes	Yes	Yes	Moderate

Questions
Q1. Is it clear in the study what is the "cause" and what is the "effect" (i.e., there is no confusion about which variable comes first)?
Q2. Were the participants included in any comparisons similar?
Q3. Were the participants included in any comparisons receiving similar treatment/care, other than the exposure or intervention of interest?
Q4. Was there a control group?
Q5. Were there multiple measurements of the outcome both pre and post the intervention/exposure?
Q6. Was follow-up complete and, if not, were differences between groups in terms of their follow-up adequately described and analyzed?
Q7. Were the outcomes of participants included in any comparisons measured in the same way?
Q8. Were outcomes measured in a reliable way?
Q9. Was appropriate statistical analysis used?

Table S4 - Case report

**Study**	**Q1**	**Q2**	**Q3**	**Q4**	**Q5**	**Q6**	**Q7**	**Q8**	**Methodological Quality**
Moshirfar et al. (2021)	Yes	Yes	Yes	Yes	Yes	Yes	Yes	Yes	High

Questions

Q1. Were patient's demographic characteristics clearly described?
Q2. Was the patient's history clearly described and presented as a timeline?
Q3. Was the current clinical condition of the patient on presentation clearly described?
Q4. Were diagnostic tests or assessment methods and the results clearly described?
Q5. Was the intervention(s) or treatment procedure(s) clearly described?
Q6. Was the post-intervention clinical condition clearly described?
Q7. Were adverse events (harms) or unanticipated events identified and described?
Q8. Does the case report provide takeaway lessons?
