# Supplementary material for: Clinical Outcomes and Complications After INTACS Implantation in Keratoconus: A Systematic Review
Source: J Clin Med. 2026 May 25;15(11):4076. doi: 10.3390/jcm15114076 (PMC13258055; doi:10.3390/jcm15114076)
Supplement: Supplementary file 1 [file jcm-15-04076-s001.zip › Supplementary Figures Complications INTACS.pdf]

Supplementary Material: Clinical Documentation of INTACS-Related Complications

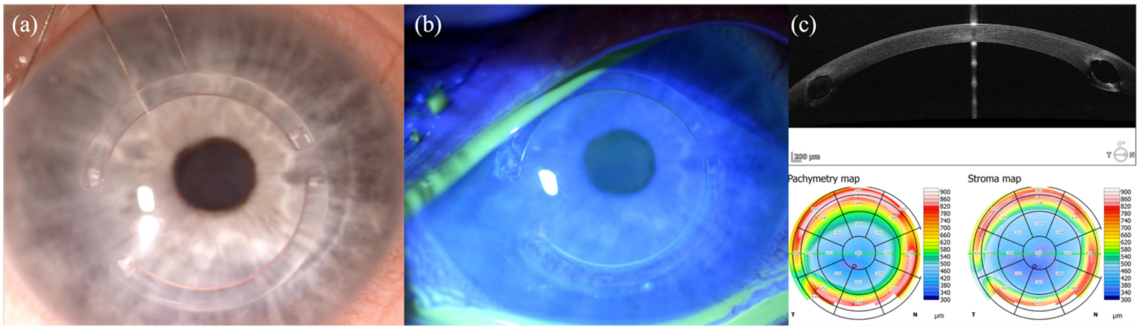

Figure S1. Multimodal imaging of the right eye with intracorneal INTACS segments. The cornea appears clear, with no visible signs of inflammation, haze, epithelial defect, or other abnormalities that could explain the patient's persistent photophobia.
(a) Slit-lamp photograph showing intracorneal INTACS segments in the corneal stroma.
(b) Fluorescein staining test demonstrating an intact corneal epithelium with no staining.
(c) Anterior segment optical coherence tomography (AS-OCT) image of the right eye demonstrating intracorneal INTACS segments implanted within the corneal stroma. The scan confirms proper intrastromal positioning and depth of the segments in the mid-peripheral cornea. Examination performed using a high-resolution spectral-domain OCT system (REVO HR, Optopol Technology).
Source: Authors' own material

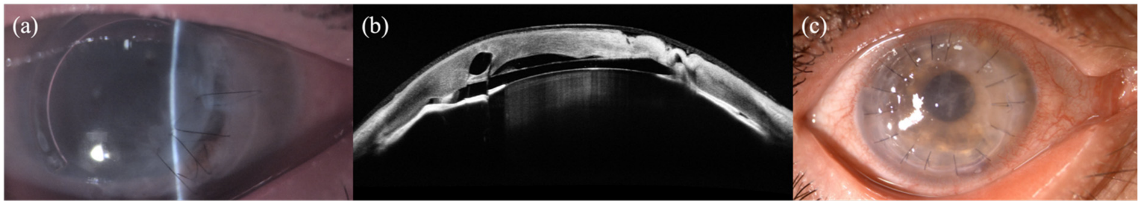

Figure S2. Clinical course following extrusion of the nasal INTACS segment in the right eye. The patient developed elevated intraocular pressure, necessitating removal of the INTACS segment. Subsequently, following a coughing episode, corneal perforation occurred at the site of prior segment implantation, likely due to localized stromal thinning. The cornea was surgically sutured; however, due to progressive structural compromise, the patient ultimately underwent penetrating keratoplasty with amniotic membrane transplantation.
(a) Postoperative slit-lamp photograph after corneal suturing, showing the site of perforation repair.
(b) Anterior segment imaging, demonstrating corneal thinning and structural irregularity at the previous INTACS location.
(c) Postoperative appearance following penetrating keratoplasty (PKP) with amniotic membrane transplantation (AMT).
Source: Authors' own material

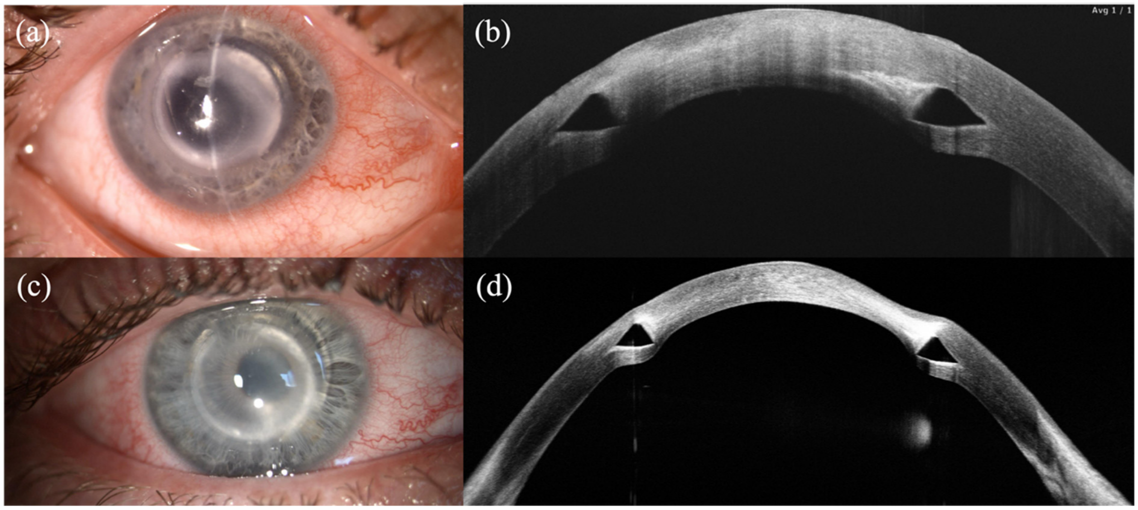

Figure S3. Clinical course of inflammatory complications following INTACS implantation in the right eye. Initial presentation demonstrated corneal edema with perisegmental inflammatory reaction. Follow-up images obtained 3 months later showed resolution of edema with residual stromal haze and localized corneal thinning at the site of prior inflammation.

(a) Slit-lamp photograph showing inflammatory reaction surrounding the INTACS segments.

(b) AS-OCT image demonstrating stromal thickening and hyperreflective changes around the INTACS segments, consistent with inflammatory involvement.

(c) Follow-up slit-lamp photograph showing resolution of corneal edema with persistent stromal haze.

(d) AS-OCT image demonstrating residual structural changes, including localized corneal thinning at the site of previous inflammation.

Source: Authors' own material.

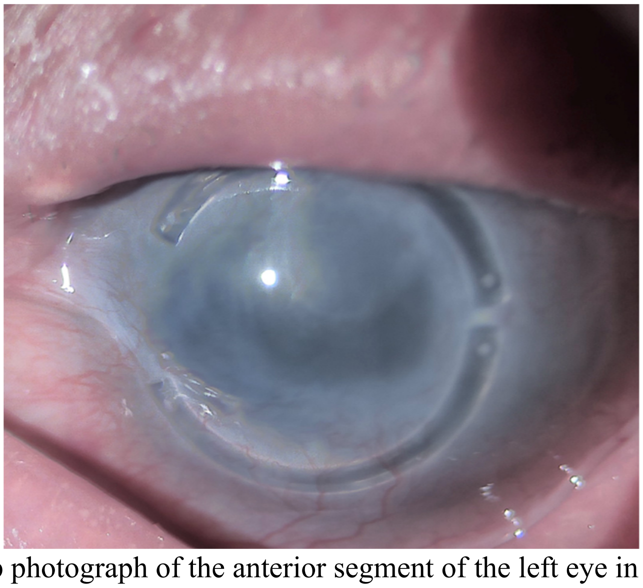

Figure S4. Slit-lamp photograph of the anterior segment of the left eye in a patient with a complex ocular history, including INTACS implantation complicated by postoperative inflammatory reaction and persistent elevation of intraocular pressure requiring surgical management with XEN gel stent implantation.

Source: Authors' own material.
