# Supplementary material for: Clinical Outcomes and Complications After INTACS Implantation in Keratoconus: A Systematic Review
Source: J Clin Med. 2026 May 25;15(11):4076. doi: 10.3390/jcm15114076 (PMC13258055; doi:10.3390/jcm15114076)
Supplement: Supplementary file 1 [file jcm-15-04076-s001.zip › Supplementary Material - Search string.pdf]

Search string:

1. **PubMed**

("intracorneal ring segments" OR INTACS OR "intrastromal corneal ring") AND (complications OR "adverse effects") AND keratoconus
Results: 40 records

2. **Scopus**

("intracorneal ring segments" OR INTACS OR "intrastromal corneal ring") AND (complications OR "adverse effects") AND keratoconus
Results: 319 records

3. **Google Scholar**

intitle:("intracorneal ring segments" OR INTACS OR "intrastromal corneal ring") AND intitle:(complications OR "adverse effects") AND keratoconus
Results: 223 records
